# Supplementary material for: Risk Assessment of Effects of Essential Oils on Honey Bees (Apis mellifera L.)
Source: Insects. 2025 Mar 14;16(3):303. doi: 10.3390/insects16030303 (PMC11942678; doi:10.3390/insects16030303)
Supplement: Supplementary file 1 [file insects-16-00303-s001.zip › insects-3479986-supplementary.pdf]

insects

MDPI

Supplementary Materials

Risk Assessment of Effects of Essential Oils on Honey Bees (*Apis mellifera* L.)

Joel Caren [1], Yu-Cheng Zhu [1,*], Quentin D. Read [2] and Yuzhe Du [1]

[1] USDA-ARS-JWDSRC, Stoneville, MS 38776, USA; joel.caren@usda.gov (J.C.); yuzhe.du@usda.gov (Y.D.)
SDA-ARS, Southeast Area, Raleigh, NC 27606, USA; quentin.read@usda.gov
* Correspondence: yc.zhu@usda.gov; Tel.: +1-662-686-5360

Table S1. Statistical data for non-significant enzyme and qPCR assay results.

Treatment	assay	method	Df	F	p	R^2
EcoTec+	EST	spray	1,19	0.1137	0.7397	0.005948
EcoTec+	EST	feed	1,16	2.94	0.1057	0.1552
EcoTec+	GST	spray	1,19	0.1954	0.6634	0.01018
EcoTec+	GST	feed	1,16	3.886	0.0662	0.1954
EcoTec+	AchE	spray	1,19	0.001363	0.9709	7.17E-05
EcoTec+	AchE	feed	1,16	4.192	0.0574	0.2076
EcoTec+	6A13	spray	1,19	1.092	0.3092	0.05434
EcoTec+	6AQ1	spray	1,19	0.6176	0.4416	0.03148
EcoTec+	6AQ1	feed	1,16	0.1052	0.7499	0.00653
EcoTec+	9Q1	spray	1,19	1.259	0.2758	0.06215
EcoTec+	9Q1	feed	1,16	0.3297	0.5738	0.02019
EcoTec+	9Q2	spray	1,19	1.45	0.2434	0.07089
EcoTec+	9Q2	feed	1,16	0.5485	0.4697	0.03314
Bisabolene	EST	spray	1,19	0.06213	0.8058	0.003259
Bisabolene	GST	feed	1,16	0.2078	0.6546	0.01282
Bisabolene	AchE	feed	1,16	3.453	0.0816	0.1775
Bisabolene	6A13	feed	1,16	1.683	0.2129	0.0952
Bisabolene	6AQ1	spray	1,19	0.4055	0.5319	0.0209
Bisabolene	6AQ1	feed	1,16	1.018	0.3281	0.05981
Bisabolene	9Q1	feed	1,16	0.3263	0.5758	0.01999
Bisabolene	9Q2	spray	1,19	0.5393	0.4717	0.0276
Bisabolene	9Q2	feed	1,16	4.479	0.0535	0.2187
Cinnamaldehyde	EST	spray	1,19	0.01167	0.9151	0.000614
Cinnamaldehyde	EST	feed	1,16	2.099	0.1667	0.116
Cinnamaldehyde	GST	spray	1,19	0.4897	0.4926	0.02512
Cinnamaldehyde	GST	feed	1,16	0.4611	0.5068	0.02801
Cinnamaldehyde	6A13	spray	1,19	1.169	0.2930	0.05798
Cinnamaldehyde	6A13	feed	1,16	1.17	0.2954	0.06814
Cinnamaldehyde	6AQ1	spray	1,19	0.03063	0.8629	0.001609
Cinnamaldehyde	6AQ1	feed	1,16	0.4812	0.4978	0.0292
Cinnamaldehyde	9Q1	spray	1,19	0.4562	0.5076	0.02345
Cinnamaldehyde	9Q2	spray	1,19	0.000265	0.9872	1.39E-05
Cinnamaldehyde	9Q2	feed	1,16	0.9436	0.3458	0.05569
Cineole	EST	spray	1,19	3.239	0.0878	0.1456
Cineole	GST	spray	1,19	0.7305	0.4034	0.03702
Cineole	GST	feed	1,16	0.07451	0.7884	0.004635
Cineole	AchE	spray	1,19	0.1871	0.6702	0.009753
Cineole	AchE	feed	1,16	0.043	0.8383	0.00268

Cineole	6A13	spray	1,22	0.3603	0.5545	0.01611
Cineole	6A13	feed	1,28	0.5141	0.4793	0.01803
Cineole	6AQ1	feed	1,28	1.778	0.1932	0.0597
Cineole	9Q2	spray	1,22	0.2618	0.6140	0.01176
Eugenol	EST	feed	1,16	3.717	0.0718	0.1885
Eugenol	GST	spray	1,19	3.177	0.0907	0.1433
Eugenol	GST	feed	1,16	1.731	0.2069	0.0976
Eugenol	AchE	feed	1,16	1.199	0.2898	0.06971
Eugenol	6A13	spray	1,19	2.733	0.1147	0.1258
Eugenol	6A13	feed	1,16	0.299	0.5920	0.01835
Eugenol	6AQ1	spray	1,19	0.08547	0.7732	0.004478
Eugenol	6AQ1	feed	1,16	0.3158	0.5819	0.01936
Eugenol	9Q1	spray	1,19	0.000608	0.9806	3.20E-05
Eugenol	9Q1	feed	1,16	0.5802	0.4573	0.03499
Eugenol	9Q2	spray	1,19	0.2619	0.6147	0.01359
Eugenol	9Q2	feed	1,16	0.005884	0.9398	0.000368

Table S2. Honey bee mortality after exposure to essential oil.

Chemical	Treatment	Concentration (mg/mL)	Exposure type	Mortality (%)	SEM	Time after exposure
Cinnamaldehyde	Control	0	contact	5	3.9	48h
Cinnamaldehyde	Cinnamaldehyde	2.1	contact	1	1	48h
Cinnamaldehyde	Cinnamaldehyde	4.3	contact	1	1	48h
Cinnamaldehyde	Cinnamaldehyde	8.55	contact	2	2	48h
Cinnamaldehyde	Cinnamaldehyde	17.1	contact	3	2	48h
Cinnamaldehyde	Cinnamaldehyde	34.2	contact	3	2	48h
Cinnamaldehyde	Cinnamaldehyde	68.5	contact	28	12.7	48h
Cinnamaldehyde	Control	0	contact	1	1	48h
Cinnamaldehyde	Cinnamaldehyde	17.1	contact	1	1	48h
Cinnamaldehyde	PBO	1%	contact	0	0	48h
Cinnamaldehyde	PBO+Cinnamaldehyde	1%+17.1	contact	4	1.9	48h
Cinnamaldehyde	TPP	1%	contact	3	3	48h
Cinnamaldehyde	TPP+Cinnamaldehyde	1%+17.1	contact	2	1.2	48h
Cinnamaldehyde	DEM	1%	contact	1	1	48h
Cinnamaldehyde	DEM+Cinnamaldehyde	1%+17.1	contact	2	2	48h
Cinnamaldehyde	Control	0	contact	0	0	48h
Cinnamaldehyde	Cinnamaldehyde	17.1	contact	0	0	48h
Cinnamaldehyde	Advise	0.13722	contact	27	5.6	48h
Cinnamaldehyde	Advise + Cinnamaldehyde	0.13722+17.1	contact	47	13.1	48h
Cinnamaldehyde	Bracket	0.0909	contact	48.8	12.1	48h
Cinnamaldehyde	Bracket + Cinnamaldehyde	0.909+17.1	contact	43.3	13.3	48h
Cinnamaldehyde	Brigade	0.13065	contact	3.8	2.4	48h
Cinnamaldehyde	Brigade + Cinnamaldehyde	0.13065+17.1	contact	35	8.9	48h
Cinnamaldehyde	Vydate	0.16156	contact	3.8	3.8	48h
Cinnamaldehyde	Vydate + Cinnamaldehyde	0.16156+17.1	contact	37.5	16.1	48h
Eugenol	Control	0	feeding	6	4	7 days
Eugenol	Eugenol	2	feeding	22	6	7 days
Eugenol	Eugenol	4	feeding	23	6.6	7 days
Eugenol	Eugenol	8	feeding	15	4.2	7 days
Eugenol	Eugenol	16	feeding	34	12.8	7 days
Eugenol	Eugenol	32	feeding	36	11.4	7 days
Eugenol	Control	0	contact	17.5	3.2	48h
Eugenol	Eugenol	67.5	contact	75	15.3	48h

Eugenol	PBO	1%	contact	13.8	6.9	48h
Eugenol	PBO+Eugenol	1%+67.5	contact	47.5	2.5	48h
Eugenol	TPP	1%	contact	7.5	3.2	48h
Eugenol	TPP+Eugenol	1%+67.5	contact	61.7	9.3	48h
Eugenol	DEM	1%	contact	0	0	48h
Eugenol	DEM+Eugenol	1%+67.5	contact	81.7	15.9	48h
Eugenol	Control	0	contact	8.8	8.8	48h
Eugenol	Eugenol	33.8	contact	20	3.5	48h
Eugenol	Advise	0.13722	contact	67.5	7.9	48h
Eugenol	Advise+Eugenol	0.13722+33.8	contact	65	5.3	48h
Eugenol	Bracket	0.0909	contact	26.3	5.9	48h
Eugenol	Bracket+Eugenol	0.909+33.8	contact	46.3	7.7	48h
Eugenol	Brigade	0.13065	contact	2.5	1.4	48h
Eugenol	Brigade+Eugenol	0.13065+33.8	contact	38.3	8.4	48h
Eugenol	Vydate	0.16156	contact	6.3	3.8	48h
Eugenol	Vydate+Eugenol	0.16156+33.8	contact	10	3.5	48h
Bisabolene	Control	0	contact	0	0	7 days
Bisabolene	Bisabolene	1.45	contact	0	0	7 days
Bisabolene	Bisabolene	2.9	contact	0	0	7 days
Bisabolene	Bisabolene	5.8	contact	0	0	7 days
Bisabolene	Bisabolene	11.6	contact	0	0	7 days
Bisabolene	Bisabolene	23.2	contact	1.3	2.5	7 days
Bisabolene	Bisabolene	46.4	contact	0	0	7 days
Bisabolene	Control	0	feeding	12.2	8.2	7 days
Bisabolene	Bisabolene	1.05	feeding	6.6	3.8	7 days
Bisabolene	Bisabolene	2.1	feeding	7.2	4.1	7 days
Bisabolene	Bisabolene	8.35	feeding	6.8	4.2	7 days
Bisabolene	Bisabolene	33.35	feeding	14	4.4	7 days
Bisabolene	Bisabolene	66.7	feeding	13	4.9	7 days
Bisabolene	Control	0	contact	1.3	2.5	48h
Bisabolene	Bisabolene	5.8	contact	1.3	2.5	48h
Bisabolene	PBO	1%	contact	0	0	48h
Bisabolene	PBO+Bisabolene	1%+5.8	contact	0	0	48h
Bisabolene	TPP	1%	contact	1.3	2.5	48h
Bisabolene	TPP+Bisabolene	1%+5.8	contact	1.3	2.5	48h
Bisabolene	DEM	1%	contact	2.5	2.9	48h
Bisabolene	DEM+Bisabolene	1%+5.8	contact	0	0	48h
Bisabolene	Control	0	contact	0	0	48h
Bisabolene	Bisabolene	5.8	contact	1.3	2.5	48h
Bisabolene	Advise	0.13722	contact	16.3	11.8	48h
Bisabolene	Advise+Bisabolene	0.13722+5.8	contact	13.8	10.3	48h
Bisabolene	Bracket	0.0909	contact	11.3	12.5	48h
Bisabolene	Bracket+Bisabolene	0.909+5.8	contact	15	23.8	48h
Bisabolene	Brigade	0.13065	contact	0	0	48h
Bisabolene	Brigade+Bisabolene	0.13065+5.8	contact	0	0	48h
Bisabolene	Vydate	0.16156	contact	3.8	2.5	48h
Bisabolene	Vydate+Bisabolene	0.16156+5.8	contact	6.3	4.8	48h
Cineole	Control	0	contact	1.3	2.5	48h
Cineole	Cineole	0.81	contact	6.3	4.8	48h
Cineole	Cineole	1.6	contact	5	4.1	48h
Cineole	Cineole	3.25	contact	3.8	4.8	48h
Cineole	Cineole	6.5	contact	2.5	5	48h
Cineole	Cineole	13	contact	5	4.1	48h

Cineole	Cineole	26	contact	0	0	48h
Cineole	Control	0	feeding	2	1.2	7 days
Cineole	Cineole	0	feeding	6	2.4	7 days
Cineole	Cineole	0.05	feeding	14	3.7	7 days
Cineole	Cineole	0.1	feeding	6	2.9	7 days
Cineole	Cineole	0.2	feeding	9	4.3	7 days
Cineole	Cineole	0.405	feeding	18	9.7	7 days
Cineole	Cineole	0.8125	feeding	9	3.7	7 days
Cineole	Cineole	3.25	feeding	16	8.4	7 days
Cineole	Control	0	contact	1.3	2.5	48h
Cineole	Cineole	3.25	contact	0	0	48h
Cineole	PBO	1%	contact	0	0	48h
Cineole	PBO+Cineole	1%+3.25	contact	5	0	48h
Cineole	TPP	1%	contact	1.3	2.5	48h
Cineole	TPP+Cineole	1%+3.25	contact	1.3	2.5	48h
Cineole	DEM	1%	contact	2.5	2.9	48h
Cineole	DEM+Cineole	1%+3.25	contact	3.8	4.8	48h
Cineole	H2O Control	0	contact	3.8	4.8	48h
Cineole	Cineole 3.25mg/ml	3.25	contact	0	0	48h
Cineole	Advise	0.13722	contact	76	7.4	48h
Cineole	Cineole+Advise	0.13722+3.25	contact	79	9.6	48h
Cineole	Bracket	0.0909	contact	0	0	48h
Cineole	Cineole+Bracket	0.909+3.25	contact	91.7	10.4	48h
Cineole	Brigade	0.13065	contact	1.3	2.5	48h
Cineole	Cineole+Brigade	0.13065+3.25	contact	0	0	48h
Cineole	Vydate	0.16156	contact	15	21.2	48h
Cineole	Cineole+Vydate	0.16156+3.25	contact	23.8	17.5	48h
EcoTec+	Control	0	contact	0	0	48h
EcoTec+	EcoTec+	0.8	contact	0	0	48h
EcoTec+	EcoTec+	1.6	contact	0	0	48h
EcoTec+	EcoTec+	3.1	contact	2.5	5	48h
EcoTec+	EcoTec+	6.2	contact	1.3	2.5	48h
EcoTec+	EcoTec+	12.4	contact	1.3	2.5	48h
EcoTec+	EcoTec+	24.8	contact	3.8	7.5	48h
EcoTec+	Control	0	feeding	19	8.2	7 days
EcoTec+	EcoTec+	0.4	feeding	22	19.9	7 days
EcoTec+	EcoTec+	0.8	feeding	12	11.5	7 days
EcoTec+	EcoTec+	3.1	feeding	29	10.8	7 days
EcoTec+	EcoTec+	12.4	feeding	38	12.5	7 days
EcoTec+	EcoTec+	24.8	feeding	35	12.7	7 days
EcoTec+	Control	0	contact	1.3	2.5	48h
EcoTec+	EcoTec+	3.1	contact	0	0	48h
EcoTec+	PBO	1%	contact	0	0	48h
EcoTec+	PBO+Ecotec	1%+3.1	contact	1.3	2.5	48h
EcoTec+	TPP	1%	contact	1.3	2.5	48h
EcoTec+	TPP+Ecotec	1%+3.1	contact	1.3	2.5	48h
EcoTec+	DEM	1%	contact	2.5	2.9	48h
EcoTec+	DEM+Ecotec	1%+3.1	contact	0	0	48h
EcoTec+	Control	0	contact	0	0	48h
EcoTec+	EcoTec+	3.1	contact	0	0	48h
EcoTec+	Advise	0.13722	contact	16.3	13.8	48h
EcoTec+	Advise+Ecotec	0.13722+3.1	contact	28.8	16	48h
EcoTec+	Bracket	0.0909	contact	21.3	39.2	48h

EcoTec+	Bracket+Ecotec	0.909+3.1	contact	10	14.1	48h
EcoTec+	Brigade	0.13065	contact	0	0	48h
EcoTec+	Brigade+Ecotec	0.13065+3.1	contact	2.5	2.9	48h
EcoTec+	Vydate	0.16156	contact	1.3	2.5	48h
EcoTec+	Vydate+Ecotec	0.16156+3.1	contact	0	0	48h
